# Blending Technology Based on HPLC Fingerprint and Nonlinear Programming to Control the Quality of Ginkgo Leaves

**DOI:** 10.3390/molecules27154733

**Published:** 2022-07-25

**Authors:** Zhe Liu, Guixin Li, Yu Zhang, Hongli Jin, Yucheng Liu, Jiatao Dong, Xiaonong Li, Yanfang Liu, Xinmiao Liang

**Affiliations:** 1 Key Laboratory of Separation Science for Analytical Chemistry, Dalian Institute of Chemical Physics, Chinese Academy of Sciences, Dalian 116023, China; liuzhe@dicp.ac.cn (Z.L.); ligx@dicp.ac.cn (G.L.); zzanwuu@dicp.ac.cn (Y.Z.); jinhl@dicp.ac.cn (H.J.); liangxm@dicp.ac.cn (X.L.); 2 University of Chinese Academy of Sciences, Beijing 100049, China; 3 Jiangxi Provincial Key Laboratory for Pharmacodynamic Material Basis of Traditional Chinese Medicine, Ganjiang Chinese Medicine Innovation Center, Nanchang 330100, China; 4 Heilongjiang ZhenBaoDao Pharmaceutical Co., Ltd., Haerbin 158400, China; ycliu2004@163.com (Y.L.); dongjiatao14@163.com (J.D.)

**Keywords:** traditional Chinese medicine quality control, natural herb blending, ginkgo leaves, high-performance liquid chromatography, stoichiometry

## Abstract

The breadth and depth of traditional Chinese medicine (TCM) applications have been expanding in recent years, yet the problem of quality control has arisen in the application process. It is essential to design an algorithm to provide blending ratios that ensure a high overall product similarity to the target with controlled deviations in individual ingredient content. We developed a new blending algorithm and scheme by comparing different samples of ginkgo leaves. High-consistency samples were used to establish the blending target, and qualified samples were used for blending. Principal component analysis (PCA) was used as the sample screening method. A nonlinear programming algorithm was applied to calculate the blending ratio under different blending constraints. In one set of calculation experiments, the result was blended by the same samples under different conditions. Its relative deviation coefficients (RDCs) were controlled within ±10%. In another set of calculations, the RDCs of more component blending by different samples were controlled within ±20%. Finally, the near-critical calculation ratio was used for the actual experiments. The experimental results met the initial setting requirements. The results show that our algorithm can flexibly control the content of TCMs. The quality control of the production process of TCMs was achieved by improving the content stability of raw materials using blending. The algorithm provides a groundbreaking idea for quality control of TCMs.

## 1. Introduction

Traditional Chinese medicine (TCM) has been used in Asia for thousands of years and is beginning to be widely accepted worldwide [1,2,3]. In recent years, Chinese medicine has demonstrated unique efficacy in the treatment of various chronic diseases. Some compounds in TCMs can induce autophagy, which has been found to cure alcoholic liver and neurodegenerative diseases [4,5,6,7,8]. Some studies have reported that certain TCMs could regulate components of apolipoprotein and key molecules involved in lipid transportation and uptake, which can help in treating conditions such as obesity and coronary heart disease [9,10,11]. TCMs have also been remarkably effective in treating patients with COVID-19 [12,13,14].

Ginkgo biloba is one of the most commonly used TCMs and has been used to treat a number of diseases, such as ischemic heart disease and cerebrovascular diseases [15,16]. Many analyses of ginkgo biloba have been established. They are mostly extracted with methanol or water or their mixtures [17]. Supercritical fluid extraction [18,19] and pressurized water extraction [20] are also possible. Separation and detection can be performed by RP-HPLC with ELSD, RI, or MS, or by GC/FID or GC/MS after silylation for routine detection [21]. In the case of evidence-based medicine, the standardized ginkgo biloba extracts (e.g., EGb 761 and Shuxuening Injection) consist of two major fractions with specific pharmacological profiles: terpene trilactones (TTL) and flavonoids [22,23]. The TTL compound class is very rare and has only been found in ginkgo biloba [24,25]. Reports have shown that ginkgolides and sesquiterpene protect mitochondria from age-related damage and improve mitochondrial function and energy metabolism [26,27,28]. There are two possible molecular mechanisms for mitochondrial protection: antagonism of platelet-activating factor receptor (PAF, 1-O-alkyl-2-acetyl-sn-glycerol-3-phosphocholine) [29,30,31] and chloride channels [32,33,34]. More than 30 genuine flavonoids are found in ginkgo biloba. Some epidemiological data suggest that flavonoids can prevent cardiovascular diseases and treat chronic venous insufficiency [35,36,37]. The positive effects resulted from antioxidative and radical scavenger effects of the flavonoids [38,39]. Ginkgo biloba has several ingredients, such as Rutin. The content of each active ingredient can have a significant effect [40]. For instance, Rutin can cause DNA damage at high doses [41]. Thus, the content consistency of TCM is important. 

However, ginkgo biloba is a natural product. Thus, the variability between samples remains high. In one study, the amount of ginkgolic acid in 11 samples of ginkgo biloba products from different sources differed by 42 times. The content of the remaining compounds could deviate by 2 to 30 times [42]. Another study of 11 ginkgo biloba products in the U.S. market showed that the difference in bilobalide content between manufacturers could be up to 20 times [43]. Many countries have proposed many quality standards to control the consistency of herbs’ content [44]. Even though all products follow the same standards, Mantle et al. found huge differences when comparing G. Biloba produced by different manufacturers [45]. As TCM is derived from agricultural products and biological organisms, its quality can be influenced by multiple factors, such as variety, origin, climate, growth years, and storage conditions [46]. Therefore, it is hard to control the consistency of the herb content at the planting stage. To clinically expand the use of herbal medicines, it is necessary to maintain their qualities. In the production of modern chemical drugs, various methods are used to manage the consistency of quality. One is the blending technique [47]. Blending technology is indispensable to ensure the stability of TCM quality. 

Only a few studies of blending technology have been reported in herbal medicine. Liu et al. presented a nonlinear least-squares-based method to blend ten batches of Fructus Gardeniae extracts. They selected seven peaks in the sample for control. The similarity between the calculation results and the target was greater than 99.9%, with less than 3% deviation after computation [48]. Tang presented a new function to blend 14 batches of Zhou prescription samples. They used Zhou’s compound medicine samples prepared with authentic medicinal materials as the target. The branch samples, after replacing some herbs, were used for mixing. The similarities between different blending schemes increased to the range of 0.9208 to 0.9797 from 0.7338 to 0.8925, and the deviation decreased to the range of 0.1549 to 0.2790 from 0.4768 to 0.6083 [49,50].

These studies were enlightening for the herb-blending process, but there are a few areas we seek to improve upon. In the mixing process, the most important thing is how to build the target. Neither of them discusses what a reasonable target should be and the process of setting them. Liu did not discuss how to establish a blending target. At the same time, the mixing process should also ensure that the mixed samples are representative. In fact, the key to blending is to take qualified samples but deviate from the target and blend them in ratios to achieve a high degree of similarity to the target with controlled content deviation. The blending process should avoid samples being too similar to the target and ensure that the samples are qualified. Tang’s target was too similar to their samples, which differed by only one herb. Secondly, these algorithms in these studies did not show flexibility for different blending constraints. Manufacturers may change the mixing constraints for other products’ needs or standards in actual production. The algorithms of Liu and Tang only performed single-condition blending, making it challenging to meet the actual needs of the manufacturer. Furthermore, these articles did not mention how to select blending samples for the blending process. In practice, not all selected samples can be blended to calculate the desired target. For example, when the content of all selected samples is less than the target, it is not possible to obtain the mixing result with equal feeding. Now, there still need to develop a blending process that establishes goals, meets multiple conditions for flexible blending, and can help users to select blending samples effectively.

In this study, we developed a new blending method based on a nonlinear optimization algorithm to meet the practical needs of manufacturers. The algorithm calculated the blending recipe to meet the overall similarity and individual content deviation requirements. Samples from different years of origin were compared to illustrate the need for blending. For the target established, high-consistency samples from the manufacturer were the target branch, and qualified samples from the market were used as blending samples. Principal component analysis (PCA) results of samples and targets were established to choose samples for mixing. Multiple sets of computational experiments were designed to demonstrate the flexibility of the nonlinear optimization algorithm for different limits of relative-deviation coefficients (RDCs) or different samples for mixing. Samples at critical conditions were chosen for the experiment, and their deviations from the computational results were analyzed. Finally, the prospect of applying algorithms in the quality control of TCMs was discussed.

## 2. Results and Discussion

### 2.1. Optimization of Chromatographic Conditions

**Figure 1:**
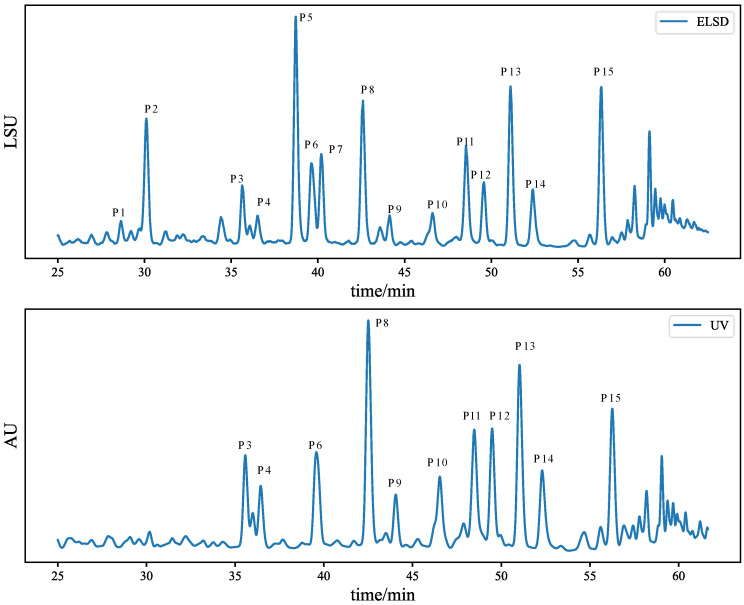
Ginkgo biloba HPLC-UV-ELSD chromatograms from 25 min to 60 min with peak designation.

To characterize the main components, the extract of the sample was analyzed by high-performance liquid chromatography with an ultraviolet detector and evaporative light-scattering detector (HPLC-UV-ELSD). The most appropriate conditions were screened out based on several chromatographic experiments, including mobile phases, gradients, flow rate, and detection wavelength. The mobile phase was composed of MeOH (A) and 1% FA (*v/v*) in H_2_O (B). The gradients of mobile phases A and B with respect to time were consistent with Table 1. The flow rate was 1.0 mL/min. The HPLC-UV-ELSD chromatograms were shown in Figure S1. After considering the quality standards of commercially available ginkgo biloba extracts such as Shuxuening injection [22,51,52], we chose the chromatograms from 25 min to 60 min and selected 15 peaks as the standard peaks to establish the standard fingerprints used in the production of ginkgo medicine. The fingerprints are shown in Figure 1. The qualitative attributions of its chromatographic peaks are shown in Table 2, identified by standard compounds shown in Table S1. Fifty-three batches of ginkgo biloba were obtained from different manufacturers, and their detailed information is listed in Table S2. The area of peak 8 in the UV chromatogram was used to calibrate all ELSD peaks. However, using semiquantitative data in blending does not require accurate compound identification. In this process, peak matching and area correction are more important than compound identification.

### 2.2. Comparison of Ginkgo Leaves from Different Sources

We compared the samples of ginkgo biloba from different origins and years, and the results are shown in Figure S2. Their average content of components was used as the standard, and the similarity and RDC of each compound were calculated, as shown in Table S3. The average of each peak was used as the unit of measurement and the number of each peak as the axis. The radar charts are shown in Figure S3. 

**Figure 2:**
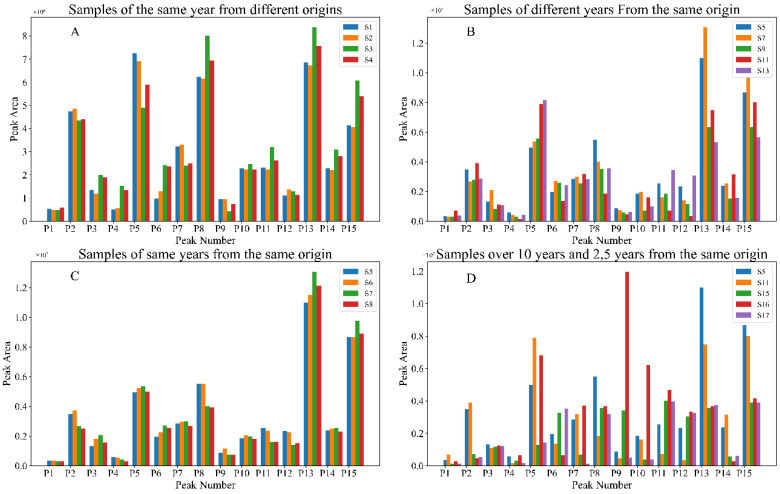
Comparison of peak areas between different samples. (**A**) Samples of the same year from different origins. (**B**) Samples of different years from the same origin. (**C**) Samples of same years from the same origin. (**D**) Samples over 10 years, 2 years and 5 years from the same origin.

To compare the samples of different years or sources, the peaks and peak areas of the samples were classified by different conditions. The results were shown in Figure 2.

Supplementary S1–S2 and S3–S4 were the samples of the same year but with different origins. From Figure 2A and Table S3, the content of P5, P8, P13, and P15 differed by more than 30%. 

Supplementary S5, S7, S9, S11, and S13 were samples of the same origin but with different growth years. Comparing Figure 2B and Table S3, the content of P13 and P15 also differed by more than 40%.

Supplementary S5–S6 and S7–S8 were samples from the same region and year. In Figure 2C and Table S3, samples of the same origin and the same year had similarities over 99% and RDCs within ±10%. 

Supplementary S15, S16, and S17 were samples with more than ten years of growth, Supplementary S5 was a sample with two years of growth, and Supplementary S11 was a sample with five years of growth, and they all have the same origin. Figure 2D and Table S3 show that the samples with more than ten years of growth period differ significantly from the other samples in composition content. The differences in the content of P2, P5, P13, P14, and P15 ranged from 200% to 300%. 

Comparing samples from different years and origins shows that the available qualified samples in the market vary greatly, which is unfavorable for quality control and subsequent quantitative research of TCMs. According to Horbowicz M. et al., this may be related to the content of methyl jasmonate in the leaves [53]. The above results also imply that it is difficult to control the quality of samples from cultivation, indicating the necessity of the blending process.

### 2.3. Establishing a Blending Target 

**Figure 3:**
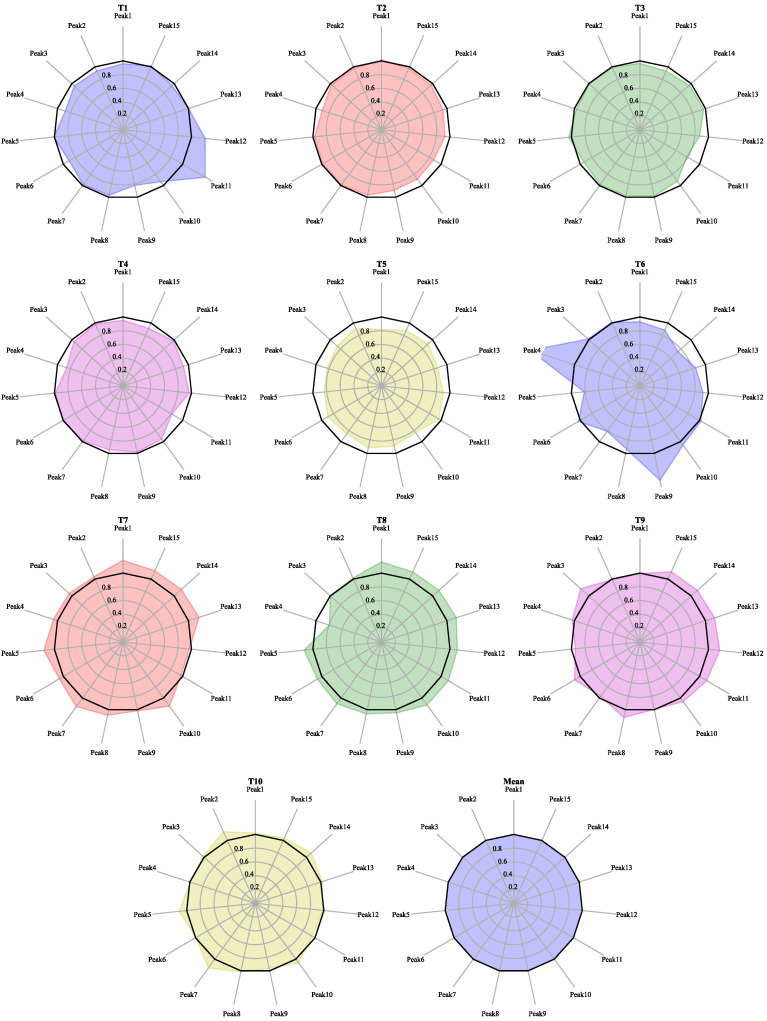
Radar diagram of ginkgo biloba target samples used for the target calculation.

High-consistency samples were used to establish the target. T1–T10, referred to as the target samples, were the high-quality branch recognized by the factory. Their chromatograms are shown in Figure S3, and radar plots are shown in Figure 3. The average value of samples T1–T10 was used as the blending target. Their similarities and RDCs in Table 3 show that the target samples have a pretty close similarity of 99.3% or higher and relatively small content differences with ±20% or lower. This is why the RDCs were controlled below ±20% in the following calculation.

It can be seen that the compositional content of the samples as targets was in good consistency. Thus, the constraints of our blending process referenced to the target samples to keep the RDCs of the blending results consistent (±20%) or better (±10%) with the target.

### 2.4. Comparing and Choosing Samples

**Figure 4:**
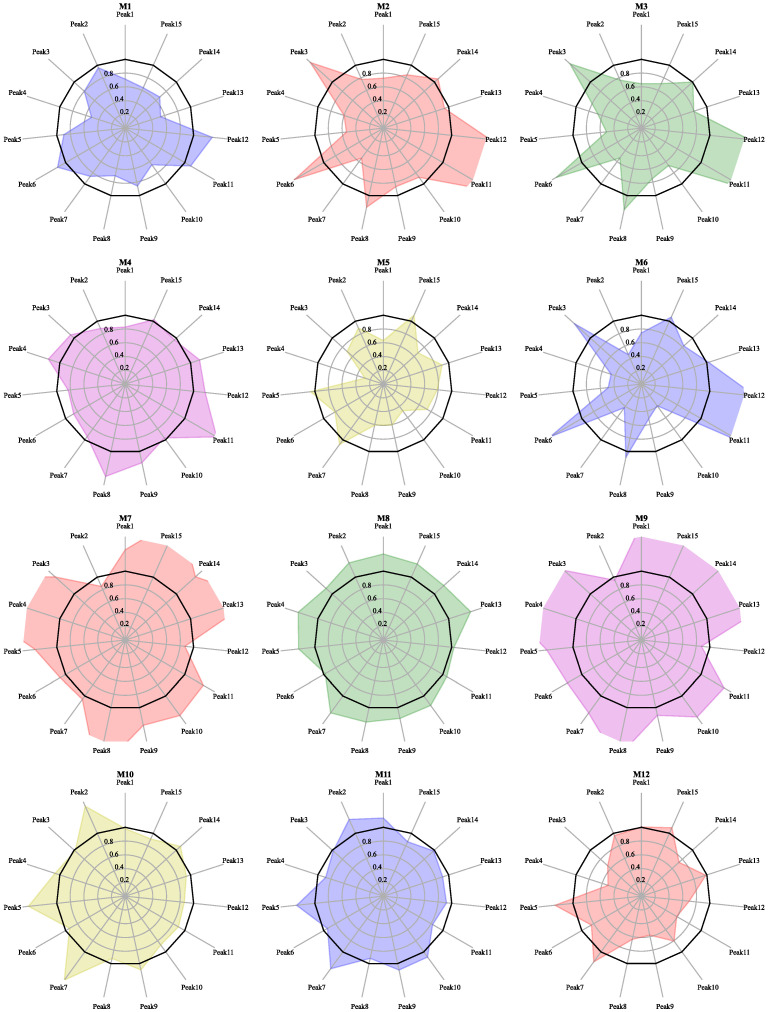
Radar diagram of qualified samples acquired in the market.

M1–M11, referred to as the blending samples for mixing, were qualified branches purchased by the factory. Their radar plots are shown in Figure 4. The similarity and RDC are shown in Table 4. The blending samples have similarities as low as about 91% and the RDC from −50% to 60%. Compared with the target samples (T1–T10), all the blending samples but M8 have lower similarities. Even in the M8 sample, which was most similar to the target, more than half of its peaks have RDCs over ±20%. 

Comparing the target samples with the qualified samples shows significant differences between even the samples of ginkgo biloba that meet the market standards. This illustrates the importance of controlling product quality with blending algorithms. 

The goal of our blending algorithm is to blend the qualified but inconsistent samples to improve consistency across batches. Thus, the significance of the blending algorithm is to provide a blending ratio that ensures a high overall fingerprint similarity between the product and the target and controls the RDCs of component contents.

**Figure 5:**
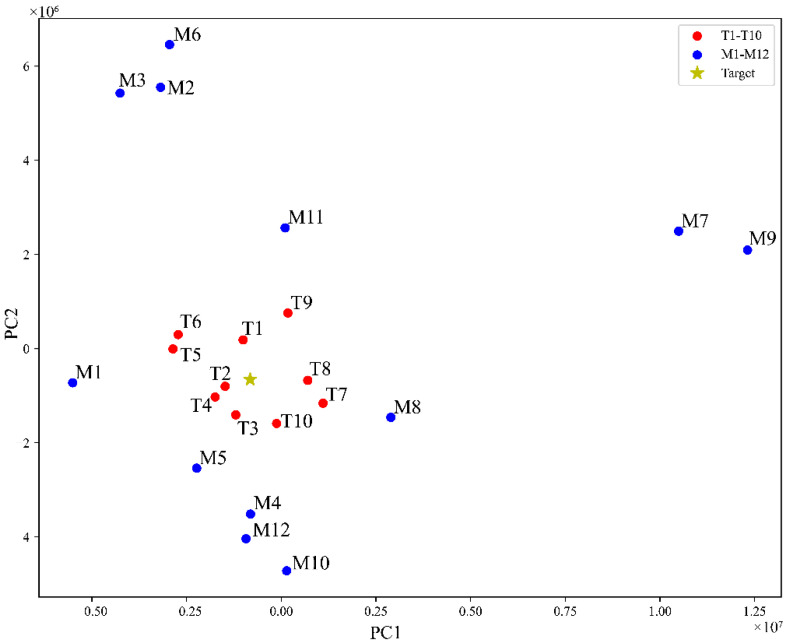
PCA results for samples M1–M11, T1–T10, and the blended target.

The PCA results for the blending samples (M1–M11), the target samples (T1–T10), and their blended target are shown in Figure 5. The PCA results show that the consistency of the target samples (T1–T10) was much higher than that of the blending samples (M1–M12). 

One great use of PCA results is effectively selecting the samples for a successful blending. If one chooses the blending samples distributed on one side around the target, the mixing will have a lower chance of meeting one given set of the relative deviation content limits as defined in Equation (7). Conversely, the selection of the blending samples symmetrically distributed around the target should be highly likely to meet the blending requirements.

### 2.5. Calculation of the Blending Method in Different Constraints with the Same Samples

**Figure 6:**
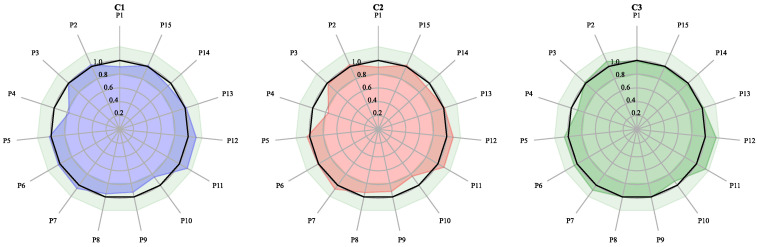
Calculated results for the same samples under different constraints. different control constraints of C1, C2 and C3 was shown in Table 5.

To verify our algorithm to perform blending under different constraints, three different blending constraints were set for mixing. The constraints 1, 2, and 3 were abbreviated as C1, C2, and C3. The RDCs of P2, P5, and P6 with mixed high- and medium-component contents were controlled within ±10% in C1. The RDCs of P11, P12, and P13 with mixed high- and medium-component contents were held within ±10% in C2. The RDCs of P1, P4, and P9 with low-component contents were controlled within ±10% in C3. All information is summarized in Table 5. Using the PCA results in Figure 5 qualified samples M1, M2, M5, and M8 were selected as blending ingredients, uniformly distributed around the target. The calculated blending-ratio results are shown in Table 5, and the projected similarity and RDCs are shown in Table 6. The radar plot of the calculation results is shown in Figure 6. The results in Figure 6 and Table 6. show that the algorithm can successfully perform blending-optimization calculations under different constraints with 99.9% or higher similarities and ±10% or lower RDCs of the selected components in all three experiments. For P4, the RDC even improved from a minimum of −45.4% to −10% (M2). 

These RDCs were lower than that in the target branch. It is difficult to make the RDCs of all peaks more inferior to the target, but it is possible to control some peaks for different application scenarios strictly. The blending results show that the blending algorithm with sample selection can lower the RDCs of chosen peaks in the blending consequence than in the target. No matter how high or low the component contents are to be controlled, with the help of the PCA results, the blending method can effectively select the blending samples and successfully calculate the blending ratio by using the blending-optimization algorithm.

### 2.6. Calculation of the Blending Method in the Same Constraints with Different Samples

**Figure 7:**
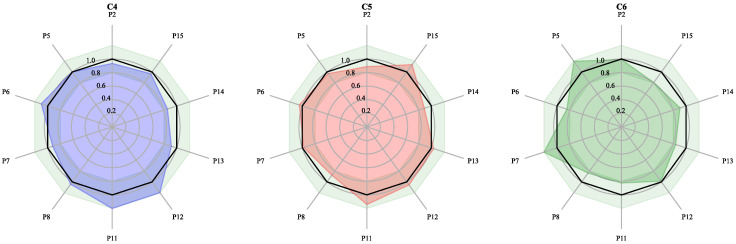
Calculated results for different samples with the same constraints. different control constraints of C4, C5 and C6 was shown in Table 7.

Using different samples for mixing under the same constraints was also verified. According to the actual needs of manufacturers, we took the similarity as the objective function and set the RDC limit less than ±20% for P2, P5, P6, P7, P8, P11, P12, P13, P14, and P15 to meet the product requirements. Using the PCA results, three sets of the blending samples were selected for mixing. The constraints 4, 5, and 6 were abbreviated as C4, C5 and C6. The mixing percentages are shown in Table 7, and their RDC and similarity are shown in Table 8. The radar plot of the calculation results is shown in Figure 7. As shown in Table 8, the blending ratios in three mixing tests satisfy their blending constraints with similarities higher than 98.5% and the RDCs within ±20%. In C4, the RDC of P7 dropped from 96% to −7.8%, and the RDC of P14 decreased from 93% to −14.3%.

The results show that the nonlinear optimization algorithm can control similarity and RDCs, working well under boundary constraints.

### 2.7. Experimental Validation of Critical Result in Calculation

**Figure 8:**
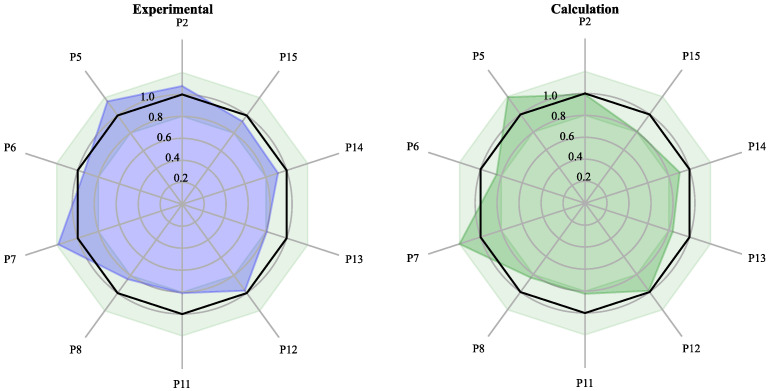
Radar diagram of calculated and experimental results.

Among results in C4–C6, the blending result C4 performs the best and C6 the worst. To verify our algorithm’s actual mixing ability and experimental accuracy, the C6 ratio was used for the experimental verification. The experimental validation results are shown in Figure 8 and Table 9. The model predicts results with similarity above 98% and RDC ±20%. The experimental results were consistent with the expected results, with content ratios between 80% and 120% and prediction deviations within the practical error range. The difference between the RDCs of calculations and experimental results was in the range of −3.6% to 12.6%. 

The experiments showed that the blending results met the constraints. The RDCs of the blending results were similar to those of the high-quality samples (T1–T10). The deviation between calculations and experiments was mainly caused by the uneven sampling of samples. If the samples can be premixed before extracting, the internal homogeneity of each sample can be improved, and the blending results can be enhanced. 

The actual experiments used the calculated marginal results, which made the experiments representative. Admittedly, the results of the actual experiments were less. Still, more experiments were conducted after adjusting the constraints to the producer’s requirements, and pilot and large-scale production were performed. The results of all experiments performed well, which will be shown in a later report.

The above results prove that the nonlinear algorithm effectively controls the similarity and RDCs of chosen peaks in actual productions.

## 3. Materials and Methods

### 3.1. Reagents and Chemicals

Standard compounds were purchased from various manufacturers or prepared in the laboratory with the detailed information shown in Table S1. Fifty-three batches of ginkgo biloba were obtained from different manufacturers, and their detailed information was listed in Table S2. Acetonitrile (HPLC grade), formic acid, and phosphoric acid (analytical purity) were purchased from Sigma-Aldrich Co. (St. Louis, MO, USA). Deionized water (18.2 MΩ·cm) was purified using a Milli-Q system (Millipore, Bedford, MA, USA). 

### 3.2. Extraction

The crushed ginkgo biloba sample was accurately weighed at 4 g and passed through a 355 μm sieve. The sample was placed in a Soxhlet extractor, then petroleum ether (30–60 °C) was added and refluxed in a 70 °C water bath for one hour. All petroleum ether was discarded in the (30–60 °C) solution, and the residue was evaporated and filtered through a paper cylinder. The mixture was dried in an oven at 60 °C and transferred to a corked conical flask. The conical flask was sonicated (power 200 W, frequency 40 kHz) for 30 min with 40 mL of methanol. The mixture was cooled, and the weight loss was made up of methanol, shaken well, and filtered. The 25 mL filtrate was measured, placed in an evaporating dish, and evaporated to dryness. Methanol was added to dissolve the residue, transferred to a 5 mL measuring flask, diluted to the methanol mark, shaken well, filtered, and finally the additional filtrate was taken.

All analyses were carried out on a Waters Alliance 2695 HPLC (Waters, Milford, MA, USA) equipped with a quaternary pump, an online vacuum degasser, and a diode-array detector. Data acquisition and processing were conducted on an Empower 3.0 workstation. Sample analysis was performed on a TNature C18 column (Waters&Acchrom-Tech, Beijing, China, 4.6 × 250 mm, 5 m). The detection wavelengths of the UV detector were set at 235 nm and 360 nm. ELSD was set as follows: gain, 100; method, heating; power, level 75%; drift tube, 75 °C; gas pressure, 30 psi.

The mobile phase was composed of MeOH (A) and 1% FA (*v/v*) in H_2_O (B) at a flow rate of 0.9 mL/min. Gradient elution is set in Table 1.

### 3.3. Blending Theory

The TCM blending-optimization model was established on the chromatographic peak area in its fingerprint. The model is based on two hypotheses: (a) The peak area is proportional to the corresponding compound concentration; (b) there are no interactions between compounds. The blending model could be expressed by following Equation (1). Equation (2) is the symbolic expression of Equation (1).

(1) $$\left[\begin{array}{cccc} s_{11} & s_{21} & \cdots & s_{1,m}\\ s_{21} & s_{22} & \cdots & s_{2,m}\\ \vdots & \vdots & \vdots & \vdots \\ s_{n1} & s_{n2} & \cdots & s_{n,m} \end{array}\right]\left[\begin{array}{c} x_{1}\\ x_{2}\\ \vdots \\ x_{n} \end{array}\right]=\left[\begin{array}{c} t_{1}\\ \vdots \\ t_{m} \end{array}\right]$$

(2) SX=T

where n is the number of samples and m is the number of components, s is the given component concentration of one sample, x is the blending ratios of one sample, and t is the given component concentration of the target. S is the matrix of component concentrations of all samples; X is the blending ratio vector, and T is the target vector of blending. Under ideal conditions, the solution to the blending problem is to find the solution vector X. However, in general, the solution vector X does not exist. Therefore, we used the cosine similarity to evaluate mixing results in Equation (3). We can convert the blending problem to maximize the objective function, which was expressed by following Equation (5).

(3) $$\mathrm{similarity}\;=\;\cos\left(\theta \right)=\frac{\mathrm{SX}\cdot T^{T}}{\left\|\mathrm{SX}\|\;\|T\right\|}$$

The blending-optimization model is aimed to maximize the overall similarity between the target and its blending result. To control the consistency of each component of the production, the RDC of content is defined as follows:

(4) $$\mathrm{RDC}{\;}_{\;i}=\frac{\left\|S_{i}X-T_{i}\right\|}{T_{i}}\;\;\;i=1,\;2,\;3,\cdots m$$

In summary, the TCM blending-optimization model can be obtained:

(5) $$\underset{X}{\max}\frac{\mathrm{SX}\cdot T^{T}}{\left\|\mathrm{SX}\|\;\|T\right\|}$$

(6) $$\{\begin{array}{c} \frac{\left\|S_{1}X-T_{1}\right\|}{T_{1}}<{\mathrm{RDClimit}}_{1}\\ \frac{\left\|S_{2}X-T_{2}\right\|}{T_{2}}<{\mathrm{RDClimit}}_{2}\\ \vdots \\ \frac{\left\|S_{i}X-T_{i}\right\|}{T_{i}}<{\mathrm{RDClimit}}_{i} \end{array}$$

The blending ratio is calculated by the COBYLA algorithm [54]. It constructs polynomial approximations L(X) to the target and constraint functions by interpolation at the vertices of simplices. It is expressed by following Equation (7).

(7) $$\hat{X}\;={\;X}_{0}-\left(\Delta \;/\;\|\underline{\nabla }L\|\right)\;\underline{\nabla }L$$

where ∆ is the trust region radius; $\underline{\nabla }L$ is the gradient of L(X); $X_{0}$ is the point of the simplex; $\hat{X}$ is the point different from all the vertices of the current simplex.

### 3.4. Blending Platform

Our blending-optimization model was based on constrained minimization of multivariate scalar functions. The calculations and preprocessing involving multimodel statistical analysis were assessed using Python® (Beaverton, OR, USA, Version 3.7.3) and SciPy® (Austin, TX, USA, Version 1.7.3). The algorithm was based on linear approximations of the objective function and RDC constraints (COBYLA) [54].

## 4. Conclusions

In this paper, we demonstrate the ability of nonlinear optimization-blending algorithms to better control the consistency of TCM well under different constraints or using different blended samples. We established blending standards by the average of high-quality samples and used qualified samples for blending. PCA results and radar plot results to screen samples during blending experiments also allow manufacturers to select batches quickly. Using the same samples for mixing under different constraints, the RDCs of the selected peaks were controlled to less than 10%, even exceeding the high-quality sample sequence, regardless of the peak area. Using different samples mixed under the same constraints, the RDCs of the selected peaks were contained to within than 20%. Despite some deviations from the calculated results, the similarity and RDCs satisfy the limiting constraints in the actual experiments. This gives an essential approach for manufacturers to control different samples under different constraints to improve the stability of product quality. This significantly improves efficiency and accuracy compared to the traditional process used in the industry. This method should be of great value and indispensable for herbal medicines with clear pharmacological activity or toxic substances.

## 5. Patents

This work has been patented with the patent number CN114580988A.

## Figures and Tables

**Table 1:** Gradient elution.

Time/min	MeOH/%	H2O (1% FA)/%
**0**	10	90
**10**	20	80
**35**	40	60
**53**	52	48
**65**	100	0
**85**	100	0

**Table 2:** Compound designation of the peaks.

Peak Label	Compounds
**P1**	Ginkgolide J
**P2**	Ginkgolide C
**P3**	Quercetin-3-O-(2,6-di-O-rhamnosyl-galactoside)
**P4**	Myricetin-3-O-rutinoside
**P5**	Ginkgolide A
**P6**	Clitorin
**P7**	Ginkgolide B
**P8**	Rutin
**P9**	Quercetin 3-O-beta-D-glucopyranosyl-(1-2)-rhamnopyranoside
**P10**	Quercetin-3-O-glucoside
**P11**	Nicotiflorin
**P12**	Narcissoside
**P13**	Quercetin-3-O-(6‴-trans-p-coumaroyl-2″-glucosyl) rhamnoside
**P14**	Kaempferol-3-O-glucosyl(1-2) rhamnoside
**P15**	Kaempferol-3-O-(6‴-trans-p-coumaroyl-2″-glucosyl) rhamnoside

**Table 3:** The similarity and RDC percent of the target samples used for target calculation.

	P1	P2	P3	P4	P5	P6	P7	P8	P9	P10	P11	P12	P13	P14	P15	Similarity
T1	−6.3	−4.8	−11.9	1	−9.8	−1.7	−2.9	−17.6	−6.7	38	19.9	0.2	−4.3	1.2	−6.3	99.5
T2	−2.2	−2.1	−9.4	−2.4	−1.5	−0.9	−3	−10.1	−11.6	−12.6	−7.3	−6.3	−3.1	−2.6	−2.2	100
T3	−0.7	−0.5	−3.1	4.1	−6.1	−1.7	−2.3	−1.6	−7.8	−21.4	−12.9	−2.9	−3.6	−6.7	−0.7	99.8
T4	−2	−4.1	−14.1	−1.3	1.2	−1.5	−5.2	−1.8	−4.5	−18	−3.2	−7.6	−1.7	−8.4	−2	99.9
T5	−8.4	−13.4	−17.1	−16.7	−9.8	−14.1	−8.2	−10.9	−16	−3.9	−10.1	−14.3	−9.2	−11.7	−8.4	99.9
T6	1	1.5	67.6	−18.7	2.9	−18.7	−6.4	39.8	6	2	−8.1	−15	−25.6	−11.4	1	99.3
T7	3.7	5.2	6.2	15.6	6.6	15.4	8.3	1.7	14.6	−2.8	−3.2	16.1	14	13.2	3.7	99.9
T8	1.8	0.2	−20.4	12.9	7.5	10.4	6.4	4.8	12.6	12	10.7	14	11.9	11.2	1.8	99.9
T9	−0.8	15.8	3.2	−5.7	9.6	−3.1	11.7	−0.6	6.5	11.6	16.3	13.9	12	11.7	−0.8	99.7
T10	13.9	2.1	−0.8	11.3	−0.5	16	1.6	−3.7	6.9	−4.8	−1.9	2.1	9.4	3.5	13.9	99.8

**Table 4:** The similarity and RDC percent of qualified samples acquired in the market.

	P1	P2	P3	P4	P5	P6	P7	P8	P9	P10	P11	P12	P13	P14	P15	Similarity
M1	−2.5	−20.1	−48.7	−9.2	13.3	−12.3	−29.6	−11.9	−33.8	13.5	31.6	−46.4	−32.4	−36.5	−2.5	97.4
M2	−21.3	42.5	−40.3	−45.4	48.8	−45.5	17	−10.8	−11.3	87.9	61.8	−7.3	7.1	−16	−21.3	93.1
M3	−22.4	39.7	−33.7	−48.2	42.9	−45.8	20.6	−23.1	−32.6	82.6	63.5	−20.7	0.4	−29.8	−22.4	92
M4	−10.2	6.7	16.8	−15.5	−13.5	−4.3	36.8	19.9	−2.7	58.5	20.9	12.7	−3.8	1.2	−10.2	98
M5	−9.4	−29.4	−72.1	7.5	−18.9	10.1	−39.1	−37.6	−51.8	−23.9	−18.4	−10.8	−32.2	8.1	−9.4	98.1
M6	−52.5	31	−53.8	−50.8	53.2	−57	8.8	−47.2	−60.8	77	72.3	1.7	−17	5.2	−52.5	91
M7	−13.5	36.6	197.4	33.4	9.1	7.7	96.6	29.7	36.1	35.3	−9.7	98	37.5	93.2	−13.5	96.2
M8	24.6	11.8	29.4	25.4	−2.5	32.5	21.2	19.2	17.8	10	6.9	32.3	18.3	19.7	24.6	99.7
M9	−3.5	51	130	40.1	24.2	32.6	76.1	14.6	38.7	43.3	−9.2	121.3	69.9	124.8	−3.5	96.3
M10	45.2	−2.1	6.6	43.2	−6.8	53.2	−7.7	12.6	−11.5	−7.9	−10.7	−7.6	8	−10.1	45.2	97.7
M11	24	−2.1	−12.4	28.2	−6.4	32.1	−8.1	12.6	10	−14.7	−4.5	−10.1	−1.6	−14.4	24	98.6
M12	−2.2	−34.1	−49.3	27.9	−16.2	19.6	−36	−40.6	−18.9	−38.9	−27.7	−2.3	−26.4	7.6	−2.2	97.2

**Table 5:** Calculated ratios of different control constraints.

	Control Peaks	RDC Limit	M1	M2	M5	M8
C1	P2, P5, P6	10%	14.1%	16.6%	27.6%	40.7%
C2	P11, P12, P13	10%	14.1%	14.1%	30.5%	40.3%
C3	P1, P4, P9	10%	25.7%	11.1%	10.1%	52.1%

**Table 6:** The similarity and RDC of calculation results.

	RDC	Control Peaks	Similarity
C1	2.5%, 2.6%, 2.7%	P2, P5, P6	99.9%
C2	10%, −0.7%, 1.1%	P11, P12, P13	99.9%
C3	−1.8%, −10%, 1%	P1, P4, P9	100%

**Table 7:** Mixing ratios of different combinations.

	Selected Samples	RDC Limit	Mixing Ratios
C4	M1:M4:M7	20%	70%:5%:25%
C5	M2:M5:M9	20%	27%:64%:9%
C6	M10:M11:M12	20%	5%:90%:5%

**Table 8:** The similarity and RDC of calculation results.

	P2	P5	P6	P7	P8	P11	P12	P13	P14	P15	Similarity
C4	−6.6	0.1	9.8	−7.8	4.2	20.0	19.6	−8.3	−14.3	−3.2	99.7
C5	−11.3	−3.0	4.7	−2.1	−12.2	14.1	5.5	3.4	−11.1	13.4	98.7
C6	−0.9	19.4	−15.4	20.0	−16.1	−17.4	−1.4	−16.1	−9.5	−19.3	98.6

**Table 9:** The similarity and RDC of calculated and experimental results.

	P2	P5	P6	P7	P8	P11	P12	P13	P14	P15	Similarity
Experimental	7.7	15.8	−6.2	18.4	−15.7	−19.1	−2.8	−19.0	−8.3	−6.7	98.8
Calculation	−0.9	19.4	−15.4	20.0	−16.1	−17.4	−1.4	−16.1	−9.5	−19.3	98.6
Deviation	8.6	−3.6	9.2	−1.6	0.4	−1.7	−1.4	−2.9	1.3	12.6	−0.2

## Data Availability

Not applicable.

## Supplementary Materials

The following supporting information can be downloaded at: https://www.mdpi.com/article/10.3390/molecules27154733/s1, Figure S1: Ginkgo biloba HPLC-UV-ELSD chromatograms; Figure S2: Ginkgo biloba HPLC-UV-ELSD chromatograms between samples of different origins and growth years; Figure S3: Radar diagram of Ginkgo biloba of samples of different origins and growth years; Figure S4: Ginkgo biloba HPLC-UV-ELSD chromatograms of samples used in the factory for actual production; Figure S5: Ginkgo biloba HPLC-UV-ELSD chromatograms of qualified samples acquired in the market; Table S1: Standard compounds information; Table S2: Sample Information; Table S3: The similarity and RDC between samples of different origins and growth years.
