# Supplementary material for: Blending Technology Based on HPLC Fingerprint and Nonlinear Programming to Control the Quality of Ginkgo Leaves
Source: Molecules. 2022 Jul 25;27(15):4733. doi: 10.3390/molecules27154733 (PMC9332425; doi:10.3390/molecules27154733)
Supplement: Supplementary file 1 [file molecules-27-04733-s001.zip › molecules-1806440-supplementary.pdf]

Support Table S1 Standard compounds information

Chemical name	Manufacturer	Purity
Ginkgolide J	Laboratory production	/
Ginkgolide A	Laboratory production	/
Ginkgolide B	Laboratory production	/
Ginkgolide C	Laboratory production	/
Quercetin-3-O-(2,6-di-O-rhamnosyl-galactoside)	Laboratory production	/
Myricetin-3-O-rutinoside	Laboratory production	/
Clitorin	CHENGDU MUST BIO-TECHNOLOGY CO., LTD	0.9999
Rutin	China National Institutes for Food and Drug Control	0.917
Queretagetin-3-O-rutinoside	Laboratory production	/
quercetin 3-O-beta-D-glucopyranosyl-(1->2)-rhamnopyranoside	CHENGDU MUST BIO-TECHNOLOGY CO., LTD	0.9995
Nicotiflorin	China National Institutes for Food and Drug Control	0.908
Narcissoside	China National Institutes for Food and Drug Control	0.931
Quercetin-3-O-(6'''-trans-p-coumaroyl-2''-glucosyl)rhamnoside	CHENGDU MUST BIO-TECHNOLOGY CO., LTD	0.98
Kaempferol-3-O-glucosyl(1-2)rhamnoside	Laboratory production	/
Kaempferol-3-O-(6'''-trans-p-coumaroyl-2''-glucosyl)rhamnoside	CHENGDU MUST BIO-TECHNOLOGY CO.,LTD	0.995

Support Table S2 Sample Information

Sample number	Sample Description	Growth year
S1	Shandong	<5
S2	Shandong	<5
S3	Shanxi	<5
S4	Shanxi	<5
S5	Pizhou	2
S6	Pizhou	2
S7	Pizhou	3
S8	Pizhou	3
S9	Pizhou	4
S10	Pizhou	4
S11	Pizhou	5

S12	Pizhou	5
S13	Pizhou	10
S14	Pizhou	10
S15	Pizhou	over 10
S16	Pizhou	over10
S17	Pizhou	over 10
T1	manufacturers	5
T2	manufacturers	5
T3	manufacturers	5
T4	manufacturers	5
T5	manufacturers	5
T6	manufacturers	5
T7	manufacturers	5
T8	manufacturers	5
M1	market	5
M2	market	5
M3	market	5
M4	market	5
M5	market	5
M6	market	5
M7	market	5
M8	market	5
M9	market	5

Support Table S3 The similarity and RDC between samples of different origins and growth years

	P1	P2	P3	P4	P5	P6	P7	P8	P9	P10	P11	P12	P13	P14	P15	Similarity
S1	134.0	157.0	102.0	99.0	127.0	46.0	123.0	141.0	60.0	126.0	90.0	62.0	92.0	115.0	66.0	96.0
S2	120.0	160.0	90.0	110.0	121.0	62.0	126.0	140.0	62.0	124.0	87.0	75.0	90.0	111.0	65.0	96.0
S3	122.0	143.0	151.0	300.0	86.0	114.0	92.0	182.0	27.0	137.0	125.0	71.0	112.0	156.0	97.0	96.0
S4	147.0	145.0	144.0	265.0	103.0	112.0	95.0	157.0	47.0	123.0	103.0	63.0	101.0	141.0	85.0	97.0
S5	86.0	115.0	99.0	111.0	87.0	92.0	109.0	125.0	57.0	103.0	100.0	128.0	147.0	119.0	138.0	98.0
S6	90.0	123.0	137.0	107.0	91.0	106.0	113.0	126.0	75.0	115.0	93.0	125.0	155.0	125.0	137.0	98.0
S7	78.0	89.0	157.0	80.0	94.0	128.0	114.0	91.0	48.0	110.0	63.0	78.0	175.0	127.0	155.0	96.0
S8	76.0	83.0	119.0	58.0	87.0	120.0	103.0	89.0	47.0	101.0	63.0	83.0	163.0	115.0	141.0	96.0
S9	75.0	92.0	63.0	61.0	97.0	122.0	97.0	80.0	38.0	40.0	73.0	64.0	85.0	76.0	100.0	99.0
S10	106.0	107.0	34.0	106.0	105.0	6.0	107.0	88.0	65.0	69.0	83.0	79.0	95.0	86.0	103.0	98.0
S11	173.0	129.0	84.0	29.0	138.0	64.0	121.0	42.0	29.0	89.0	28.0	18.0	100.0	159.0	127.0	95.0

S12	187.0	138.0	101.0	12.0	143.0	123.0	134.0	46.0	29.0	82.0	56.0	50.0	114.0	158.0	140.0	96.0
S13	92.0	95.0	82.0	79.0	142.0	114.0	108.0	81.0	39.0	55.0	134.0	170.0	71.0	79.0	90.0	96.0
S14	80.0	67.0	63.0	58.0	113.0	139.0	85.0	76.0	53.0	39.0	106.0	102.0	51.0	62.0	67.0	95.0
S15	33.0	24.0	89.0	65.0	22.0	154.0	27.0	81.0	220.0	22.0	157.0	168.0	48.0	29.0	62.0	83.0
S16	71.0	15.0	95.0	126.0	119.0	31.0	141.0	83.0	771.0	344.0	183.0	184.0	49.0	13.0	66.0	69.0
S17	31.0	18.0	90.0	35.0	25.0	167.0	5.0	73.0	33.0	22.0	155.0	180.0	50.0	31.0	62.0	84.0

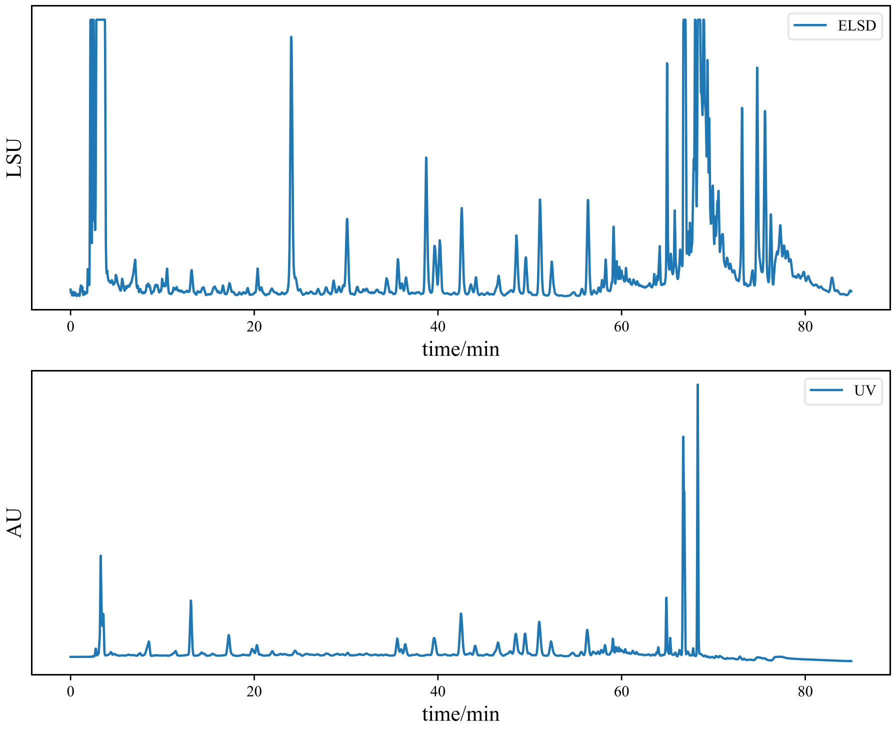

Support Figure S1 Ginkgo biloba HPLC-UV-ELSD chromatograms

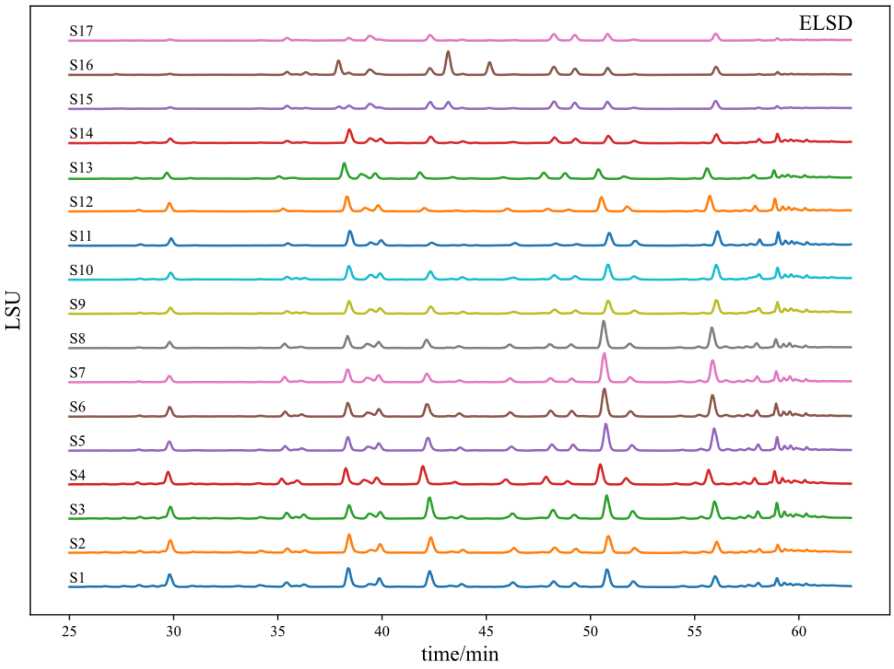

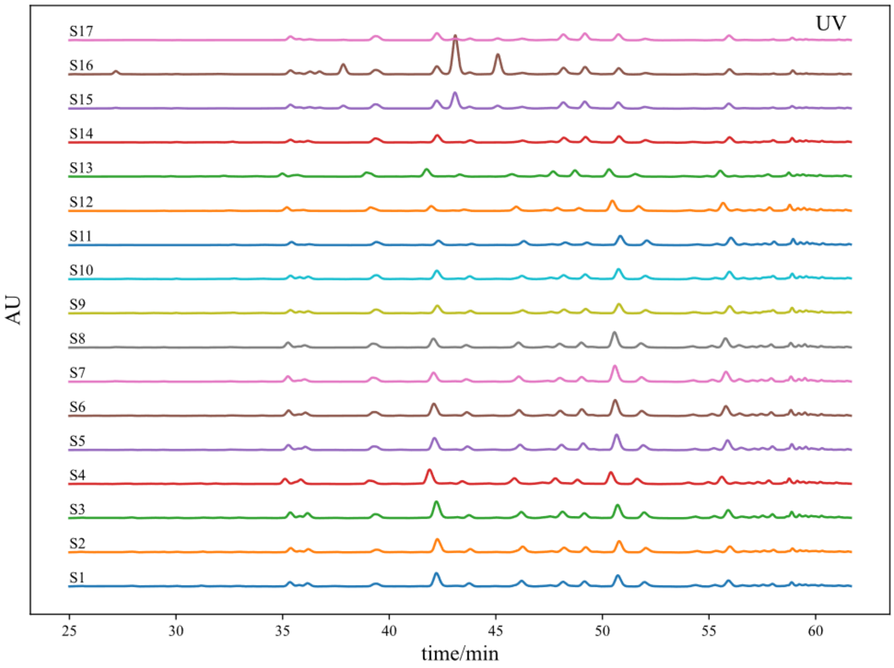

Support Figure S2 Ginkgo biloba HPLC-UV-ELSD chromatograms between samples of different origins and growth years

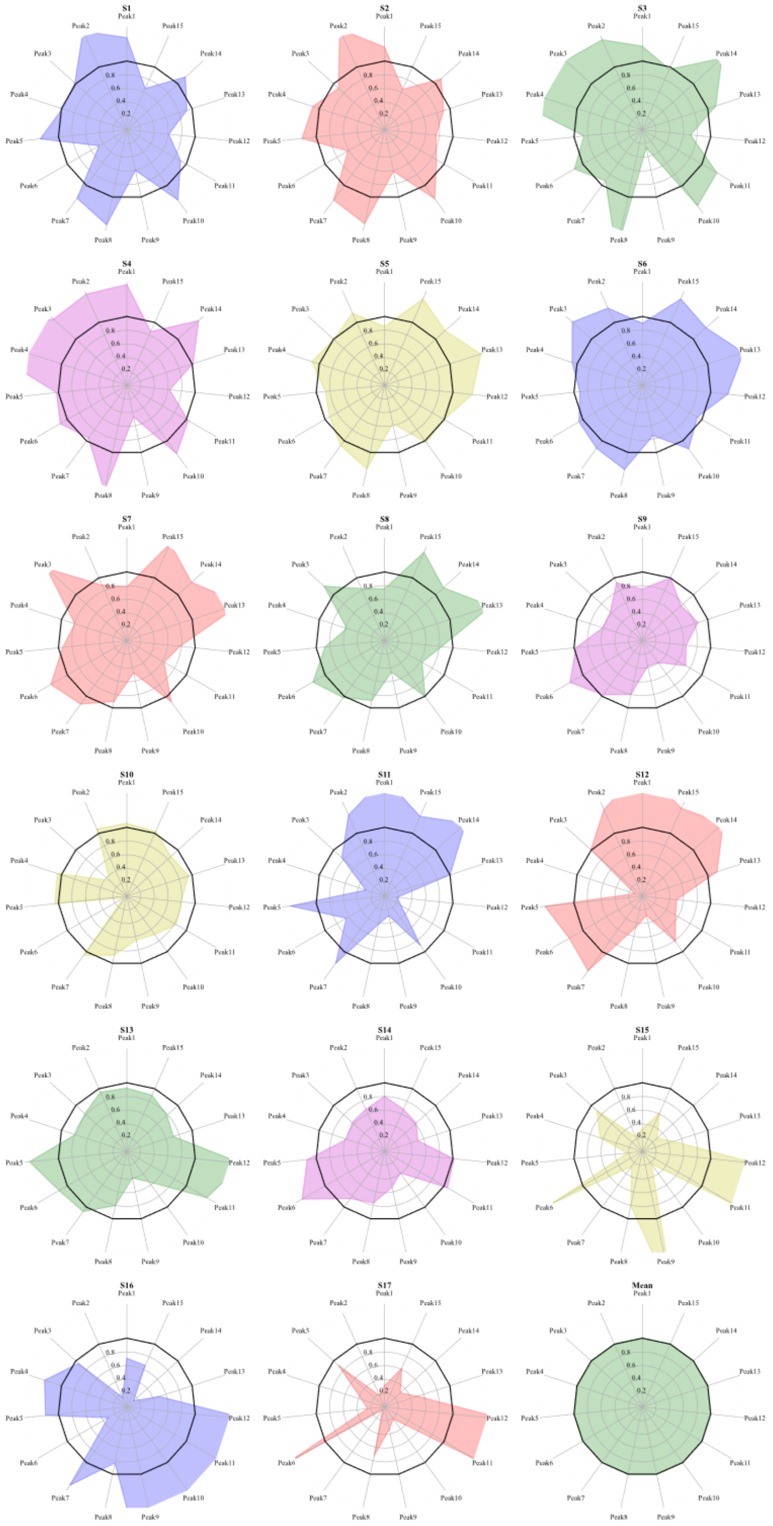

Support Figure S3 Radar diagram of Ginkgo biloba of samples of different origins and growth years

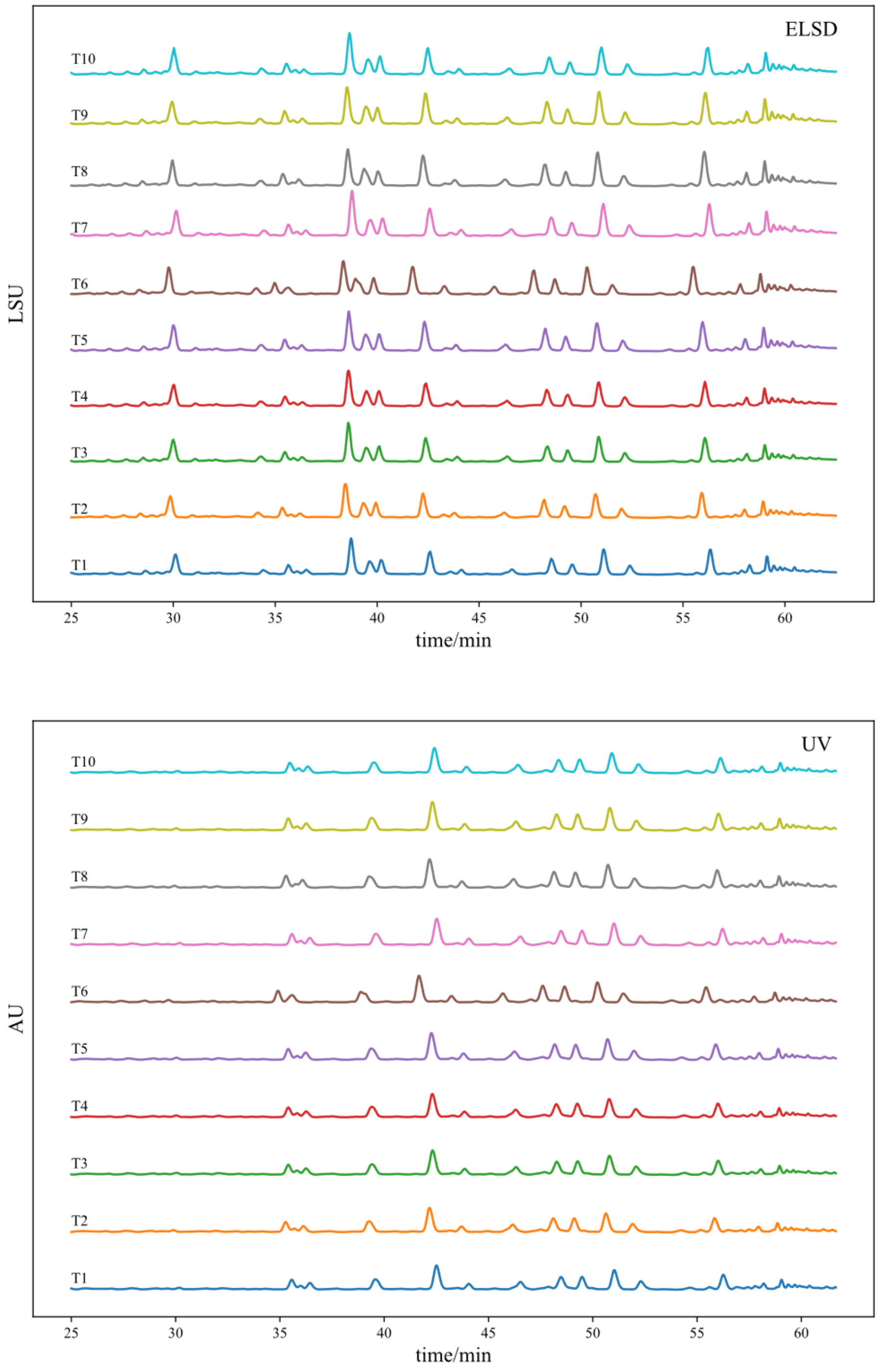

Support Figure S4 Ginkgo biloba HPLC-UV-ELSD chromatograms of samples used in the factory for actual production.

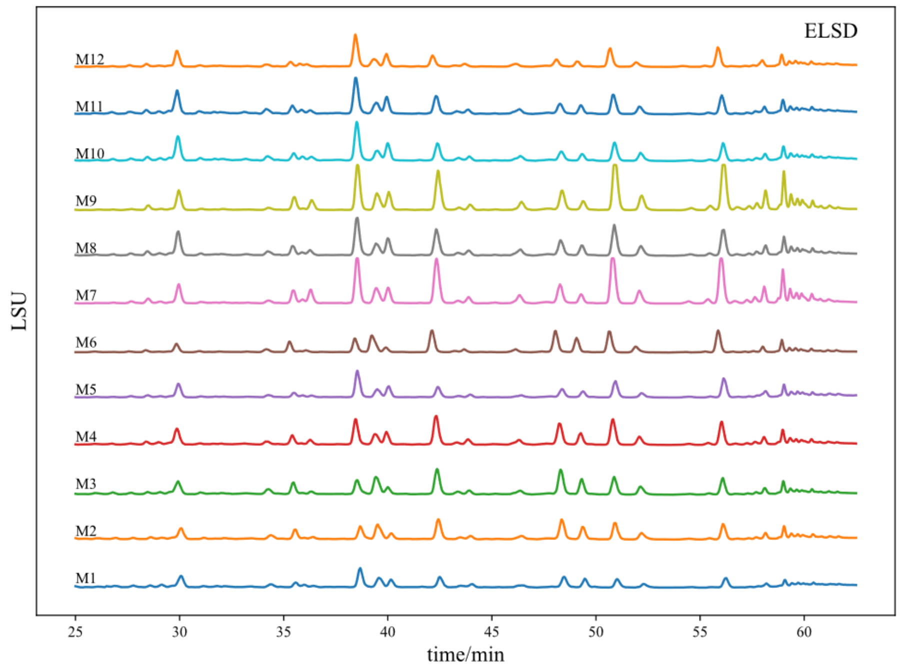

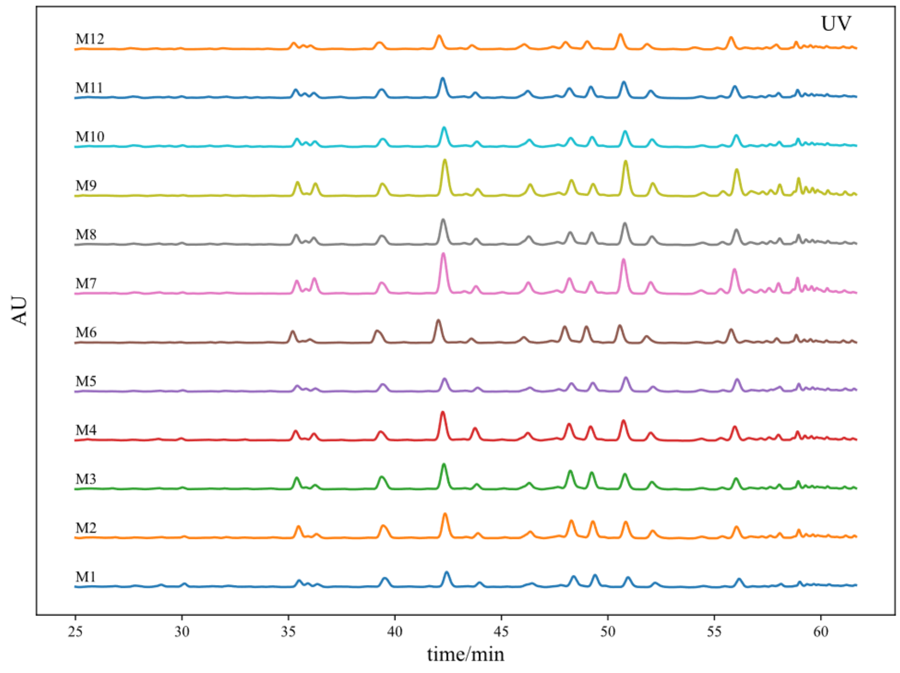

Support Figure S5 Ginkgo biloba HPLC-UV-ELSD chromatograms of qualified samples acquired in the market.
